# Abnormalities of Resting-State Electroencephalographic Microstate in Rapid Eye Movement Sleep Behavior Disorder

**DOI:** 10.3389/fnhum.2021.728405

**Published:** 2021-10-22

**Authors:** Anjiao Peng, Ruien Wang, Jiamin Huang, Haiyan Wu, Lei Chen

**Affiliations:** ^1^Department of Neurology and Joint Research Institute of Altitude Health, West China Hospital, Sichuan University, Chengdu, China; ^2^Centre for Cognitive and Brain Sciences and Department of Psychology, University of Macau, Macau SAR, China

**Keywords:** electroencephalogram, REM sleep behavior disorder, microstate, biomarker, neurodegeneration diseases

## Abstract

**Objective:** Rapid eye movement (REM) sleep behavior disorder (RBD) is a disease characterized by dream enacting behavior and is now commonly believed to be a harbinger to alpha-synucleinopathy diseases such as dementia with Lewy bodies, Parkinson's disease, and multiple system atrophy. The aim of this study was to explore the quasi-stable topological structure of the brain in RBD by analyzing resting-state electroencephalography (EEG) microstates.

**Methods:** We enrolled 22 participants with RBD and 46 healthy controls (HCs) with age and gender-matched. After the resting-state EEG recordings were acquired, EEG microstate features were analyzed to assess the functional networks of all participants.

**Results:** Significant differences in the brain topological structure and temporal characteristics of sub-second brain activity were identified between the RBD and HCs. The RBD group had a shorter average duration of microstate A and microstate D when compared with HCs, and microstate B contributed more, while microstate D contributed significantly less to the RBD group. Furthermore, the average duration and proportion of microstate D were negatively correlated with the RBD questionnaire Hong Kong (RBDQ-HK) score.

**Conclusion:** The result of this study indicates that the microstate dynamics is disturbed in RBD, which might jeopardize the flexibility and adaptability of the brain. Microstates are potential biomarkers to explore the early electrophysiological abnormality of alpha-synucleinopathy diseases.

## Highlights

- The brain topological structure and temporal characteristics of sub-second brain activity were altered in RBD.
- The average duration and proportion of microstate D were negatively correlated with the severity of the symptom.
- Microstates are potential biomarkers to explore the early electrophysiological features of neurodegenerative diseases.

## Introduction

Rapid eye movement (REM) sleep behavior disorder (RBD) is a parasomnia characterized by muscle atonia during REM sleep, which is usually accompanied by vivid dream enactment (Postuma and Berg, 2019). Previous studies showed that nearly 80% of RBD individuals could develop Parkinson's disease (PD) and other alpha-synucleinopathy diseases within 10–15 years (Iranzo et al., 2014; Postuma and Berg, 2019). Therefore, RBD is now widely recognized as a prodrome of alpha-synucleinopathy diseases and is ideal for exploring early pathological changes of these diseases (Postuma et al., 2009; Perkins et al., 2021).

Electroencephalography (EEG) microstates are defined as short periods (80–120 ms) during which the EEG scalp topography remains quasi-stable, meaning that the global topography is fixed but strength might vary and polarity invert (Strik and Lehmann, 1993). These microstates have been considered as the building blocks of information processing in the human brain, disruption of which could significantly influence cognitive function (Schumacher et al., 2019). The investigation of temporal aspects of microstate sequences also provides us with essential information on the dynamic repertoire across different timescales of the brain (Van de Ville et al., 2010).

For now, four EEG microstates have been widely identified to be closely correlated with resting-state networks by using simultaneous functional MRI (fMRI) (Lehmann et al., 2005; Michel and Koenig, 2018). Microstate A is interpreted as an auditory or sensorimotor system, which is significantly correlated with the changes in negative blood-oxygen-level dependence (BOLD) activation of the temporal-parietal cortex (Britz et al., 2010; Gschwind et al., 2016). Microstate B is regarded as a visual network, which mainly correlates with the BOLD changes in striate and extrastriate cortices and the negative BOLD activation of the occipital cortex (Michel and Koenig, 2018). Microstate C is construed as the salience network, associated with positive BOLD activation in frontal, anterior cingulate cortex, and anterior insular cortices (Gschwind et al., 2016; Michel and Koenig, 2018). Microstate D is interpreted as the attention network, mainly reflecting the activation of the right inferior parietal, right middle and superior frontal gyri, and right insula (Yuan et al., 2012; Custo et al., 2017).

As an ideal way to study the momentary local states and interaction between networks of the brain, microstates have been accumulating attention in recent years (Khanna et al., 2015; Serrano et al., 2018; Chu et al., 2020). It has been widely studied in neuropsychiatric disorders such as schizophrenia (Koenig et al., 1999), depression (Murphy et al., 2020), Alzheimer's disease (Tait et al., 2020), PD (Chu et al., 2020), and Lewy body dementia (Schumacher et al., 2019). Previous studies found that individuals with PD showed many altered states of microstates such as longer duration and more occurrences of specific types of microstates, which were closely associated with motor function and recognition level (Chu et al., 2020). The duration and occurrence of microstates were also abnormal in individuals with Lewy body dementia (Schumacher et al., 2019). This study indicates that brain networks were altered in alpha-synucleinopathy diseases. As a predictor of alpha-synucleinopathy diseases, stronger functional connectivity between the left thalamus and occipital regions was also found in RBD using fMRI (Byun et al., 2020). In fact, a thorough understanding of the spatiotemporal dynamics of the large-scale neural network is helpful for identifying the abnormality of the RBD and would provide us with a novel perspective to understand the early pathogenesis of neurodegenerative diseases. However, EEG microstates have not been studied in RBD hitherto. The main goal of this study was to analyze the characteristics of resting-state EEG microstates in individuals with RBD, compared with healthy controls (HCs), and at the same time, to explore the potential validity of EEG microstate in assessing early stage alpha-synucleinopathy diseases and the potential clinical value of resting-state microstate as an early biomarker for the diagnosis of RBD.

## Methods

### Study Population

With approval from the Ethics Committee of West China Hospital, Sichuan University, 22 participants with RBD and 46 HCs with age and gender-matched were enrolled with informed consents. All participants were free of diagnosis of PD, Alzheimer's disease, traumatic brain injury, stroke, depression, anxiety, schizophrenia, or any other neuropsychiatric disorder. No participant was taking any medicine. Individuals with RBD were first screened based on the Chinese RBD questionnaire Hong Kong (RBDQ-HK), which was verified to have high sensitivity (82.2–85%), specificity (81–86.9%), internal consistency, and test-retest reliability in the Chinese population (Li et al., 2010; Shen et al., 2014). The total score ranges from 0 to 100, and a score of 17 or higher was considered as candidates of RBD. When RBD was suspected, the diagnosis would be further confirmed by neurologists. Results from a previous study showed that the clinical diagnosis by clinicians could provide a good sensitivity (100%) and specificity (99.6%) in diagnosing RBD (Eisensehr et al., 2001). Thus, although the diagnoses of RBD in this study were not confirmed by polysomnography, we believed that the vast majority of participants were correctly grouped.

### EEG Recording and Task Procedure

Participants were seated in a quiet room for exams. The data of a 5-min resting-state EEG were obtained from all participants using 64 Ag/AgCl electrodes (Brain Products, Munich, Germany) placed following the 10/20 system with impedance <10 kΩ. An actiCHamp amplifier (BrainVision system amplifier; Brain Products, Munich, Germany) was used to amplify and collect the EEG data at a sampling rate of 1,000 Hz. Participants were instructed to fixate their sight at the center of the screen and try to avoid body movement during the EEG recording.

### EEG Data Pre-processing

EEGLAB toolbox (Delorme and Makeig, 2004) in MATLAB (R2017a, MathWorks, Natick, MA, USA) was used to process the EEG data offline. Data were first down-sampled to 250 Hz, filtered with a 1–40 Hz filter, and then filtered with a 50 Hz notch filter for removing line noise. Then, a clean raw data plug-in of EEGLAB was used for bad channel detection and removal. Maximum flatline duration was set at 5 s for the detail parameter setting, baseline noise criterion was set at 4 SDs, and the minimum acceptable correlation with nearby channels was set at 0.8. All the removed channels were interpolated by using the spherical method, and all channels were referenced to the average reference after interpolation. As for the proportion of the bad channel rejection and interpolation, the overall average proportion of bad channel rejection is 18.88 (SD = 8.80); specifically, 19.81 (SD = 9.21) for the RBD group and 18.39 (SD = 8.73) for the HC group. To further minimize artifacts, the artifact subspace reconstruction method (Mullen et al., 2015) was used to correct the EEG data, and the maximum acceptable 0.5-s window was set at 20 SDs.

After preliminary data processing, the continuous data were segmented into epochs of 2 s for further microstate analysis. Independent component analysis (ICA) was implemented by using the runica function in EEGLAB to remove the artifact caused by ocular movement, cardio activity, and muscle movement. A semiautomatic manner (i.e., ICLabel plug-in) was used to remove the artifact, and visual inspection was used for further removal.

### Microstate Analysis

Microstate analysis was performed using the microstate plug-in of EEGLAB developed by Thomas Koenig (Koenig, 2021). First, at the individual level, we calculated the global field power (GFP) across the whole channels and the microstate segmentation by using the Atomize and Agglomerate Hierarchical Clustering (AAHC) method. Polarity was ignored during the microstate analysis. After obtaining the microstate segmentation of each participant, we calculated a mean microstate segmentation of each group as templates. The original individual successive EEG series were then assigned into four classic microstate maps (i.e., A, B, C, and D classes). For each microstate, the following parameters were extracted for further analysis: the mean duration (i.e., the duration of each microstate status with a unit of milliseconds), occurrence rate per second (i.e., times a certain microstate status would occur in a second), the time proportion of a microstate status (i.e., the percentage of each microstate status in the segmentation), and the percentage of transitions between microstate status.

### Statistical Analysis

Continuous variables for clinical data were described as mean and SD and calculated using the *t*-test. Categorical variables were expressed as frequencies and percentages and calculated using the chi-square test. The mean and SE of mean were calculated for the microstate global explained variance (GEV) in two groups. Shapiro-Wilk test was used to examine the normality of the microstate parameters, and the difference of microstate parameters between two groups was compared by using the independent *t*-test if the data conform to normality. Otherwise, the Mann-Whitney *U* test was used. Pearson correlation analyses and linear regression were adopted to analyze the relationship between the microstate parameters and RBDQ-HK scores, and ANCOVA was further used to test the effects of grouping on the relationship between microstate parameters and RBDQ-HK score. Statistical analysis was performed using R language (version 4.0.5, R Core Team, Vienna, Austria) and SPSS version 25 (version 25, IBM Inc., Armonk, NY, USA).

## Results

### Demographic Characteristics and Clinical Assessment Data

A total of 22 participants with RBD and 46 HCs were included. The mean age was 56.9 ± 6.2 years for the RBD group and 55.0 ± 4.5 years for HCs (*p* = 0.165). The mean score of the Montreal Cognitive Assessment (MoCA) was 25.27 ± 5.0 for the RBD group and 26.26 ± 3.2 for HCs (*p* = 0.328). Gender, educational level, and marital status were compared between the two groups (Table 1).

**Table 1:** Demographic features for participants with RBD or healthy controls.

	**Controls (*n* = 46)**	**RBD (*n* = 22)**	***P-*value**
Age (mean ± SD, years)	55.0 ± 4.5	56.9 ± 6.2	0.165
Gender (female, %)	35 (76.0%)	16 (72.7%)	0.772
Educational level	10.3 ± 3.2	10.6 ± 3.9	0.674
Marital status (married, %)	39 (84.8%)	20 (90.9%)	0.707
MoCA	26.26 ± 3.2	25.27 ± 5.0	0.328

*MoCA, Montreal Cognitive Assessment; RBD, REM sleep behavior disorder; SD, standard deviation*.

### Microstate Topographies Were Altered in RBD

**Figure 1:**
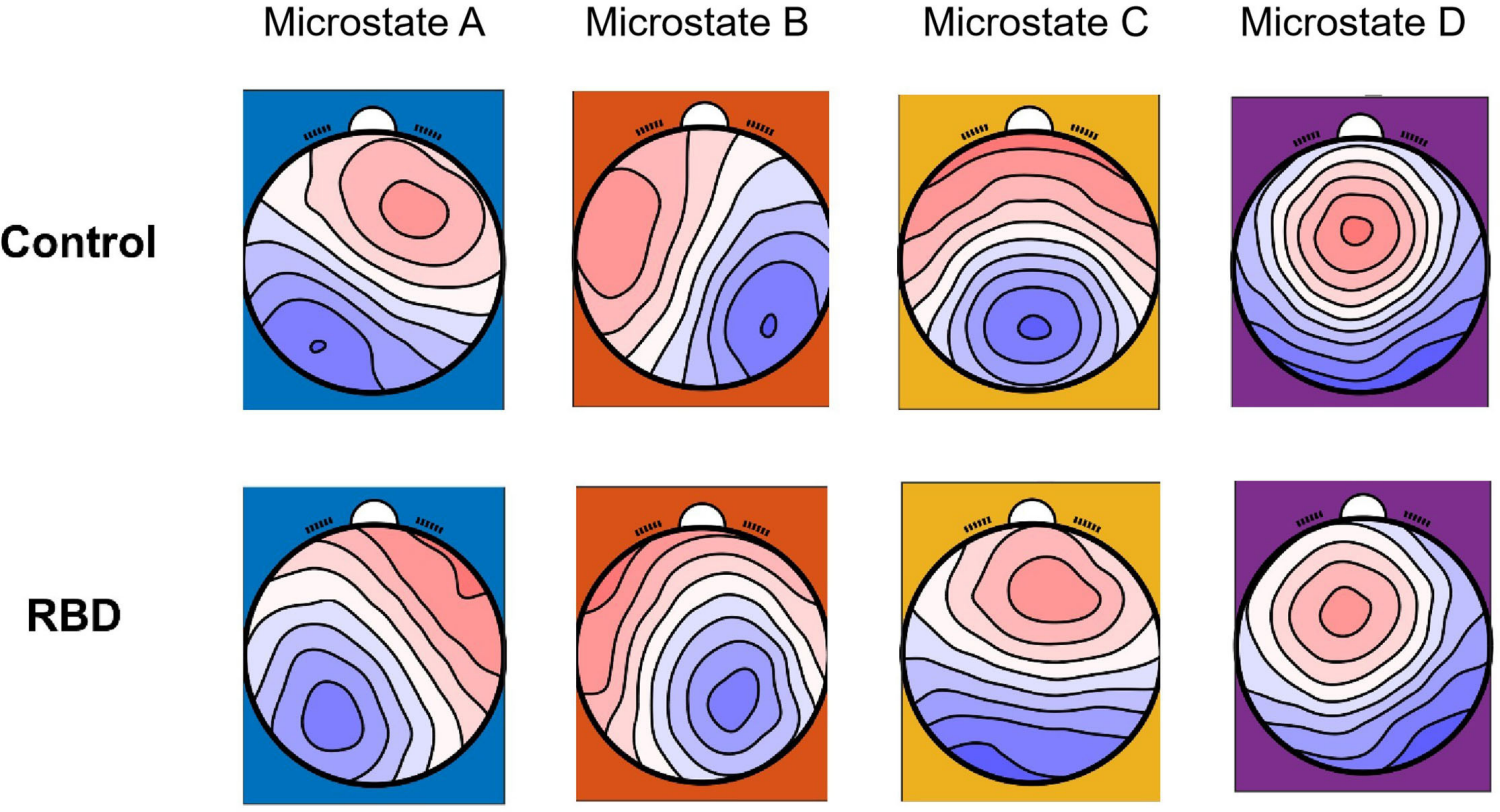
Microstate topographies of participants with RBD and healthy controls (HCs).

Four classes of mean microstate topographies were obtained from both groups (Figure 1). The mean microstate maps of each group were used as the template for further analysis, and the GEV of each template was consistent between the two groups with 0.725 (SE of mean, SEM = 0.004) for the RBD group and 0.716 (SEM = 0.006) for HCs. The results showed that the topographies were altered in the RBD group compared with HCs, characterized by more diffused center microstate B on the left frontal region and more focused center microstate C on the middle frontal-central region. Besides, the center of microstate D was more lateralized to the left frontal-central area in RBD.

### Abnormalities of Microstate Parameters in RBD

**Figure 2:**
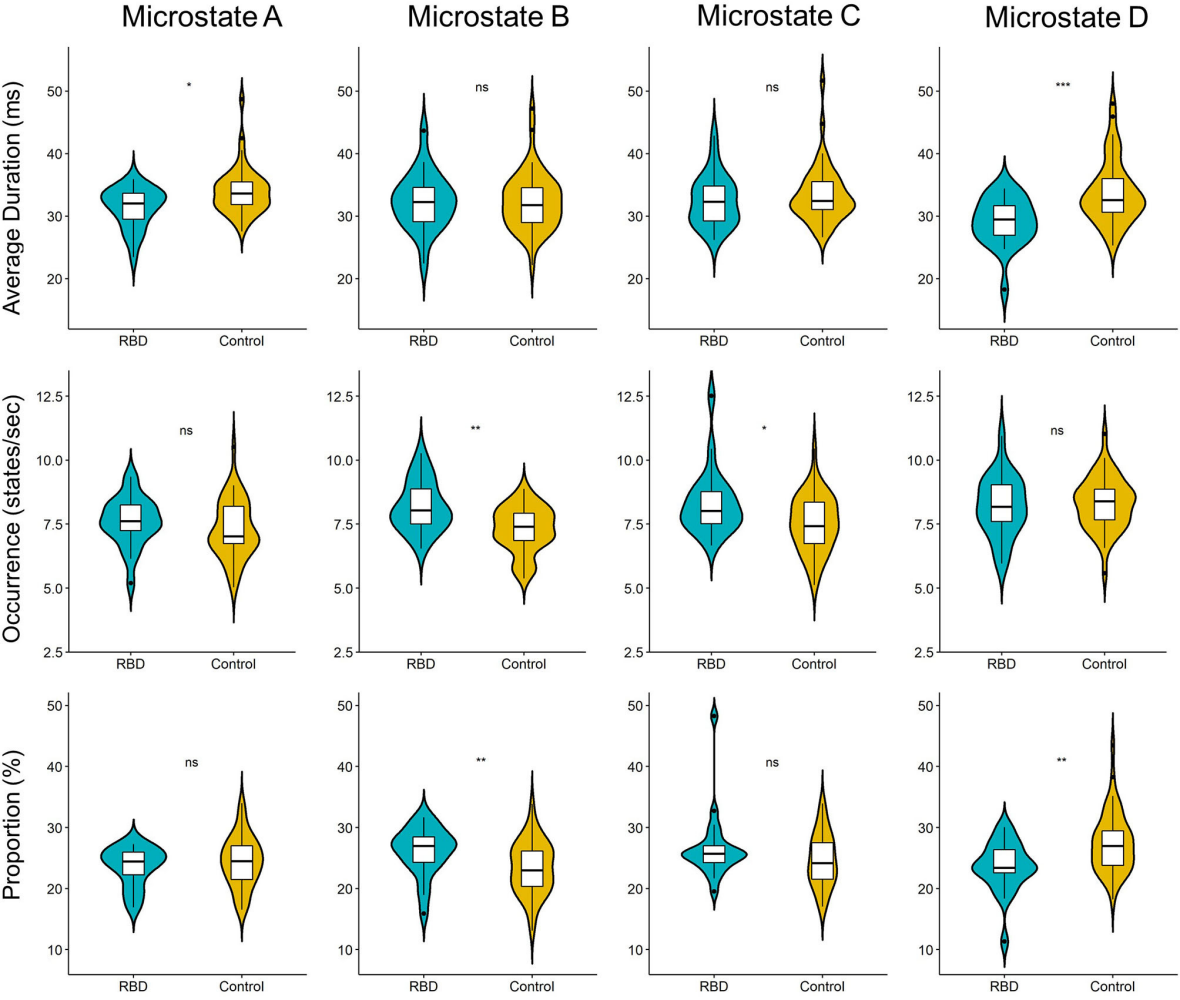
Violin plot of the microstate parameters of each microstate between participants with RBD and HCs. The RBD group had a shorter average duration of microstate A (*p* = 0.013) and microstate D (*p* < 0.001). Regarding the occurrence, the RBD group produced more microstate B (*p* = 0.002) and microstate C (*p* = 0.017) per second than HCs. Microstate B contributed more (*p* = 0.007), while microstate D contribute significantly less (*p* = 0.003) in the RBD group when compared with HCs (ns, not significant; *p < 0.05, **p < 0.01, and ***p < 0.001).

Three parameters (i.e., average duration, occurrence, and proportion) were calculated for each EEG microstate. The results showed that compared with HCs, the RBD group had a shorter average duration of microstate A (*M*_HC_ 33.98 vs. *M*_RBD_ 31.38; *p* = 0.013) and microstate D (*M*_HC_ 33.75 vs. *M*_RBD_ 29.13; *p* < 0.001). Regarding the occurrence, the RBD group produced more microstate B (*M*_HC_ 7.29 vs. *M*_RBD_ 8.19; *p* = 0.002) and microstate C (*M*_HC_ 7.53 vs. *M*_RBD_ 8.33; *p* = 0.017) per second than HCs. Furthermore, the results showed that microstate B contributed more (*M*_HC_ 23.37 vs. *M*_RBD_ 26.11; *p* = 0.007), while microstate D contributed significantly less (*M*_HC_ 27.48 vs. *M*_RBD_ 23.58; *p* = 0.003) in the RBD group than HCs (Figure 2).

**Figure 3:**
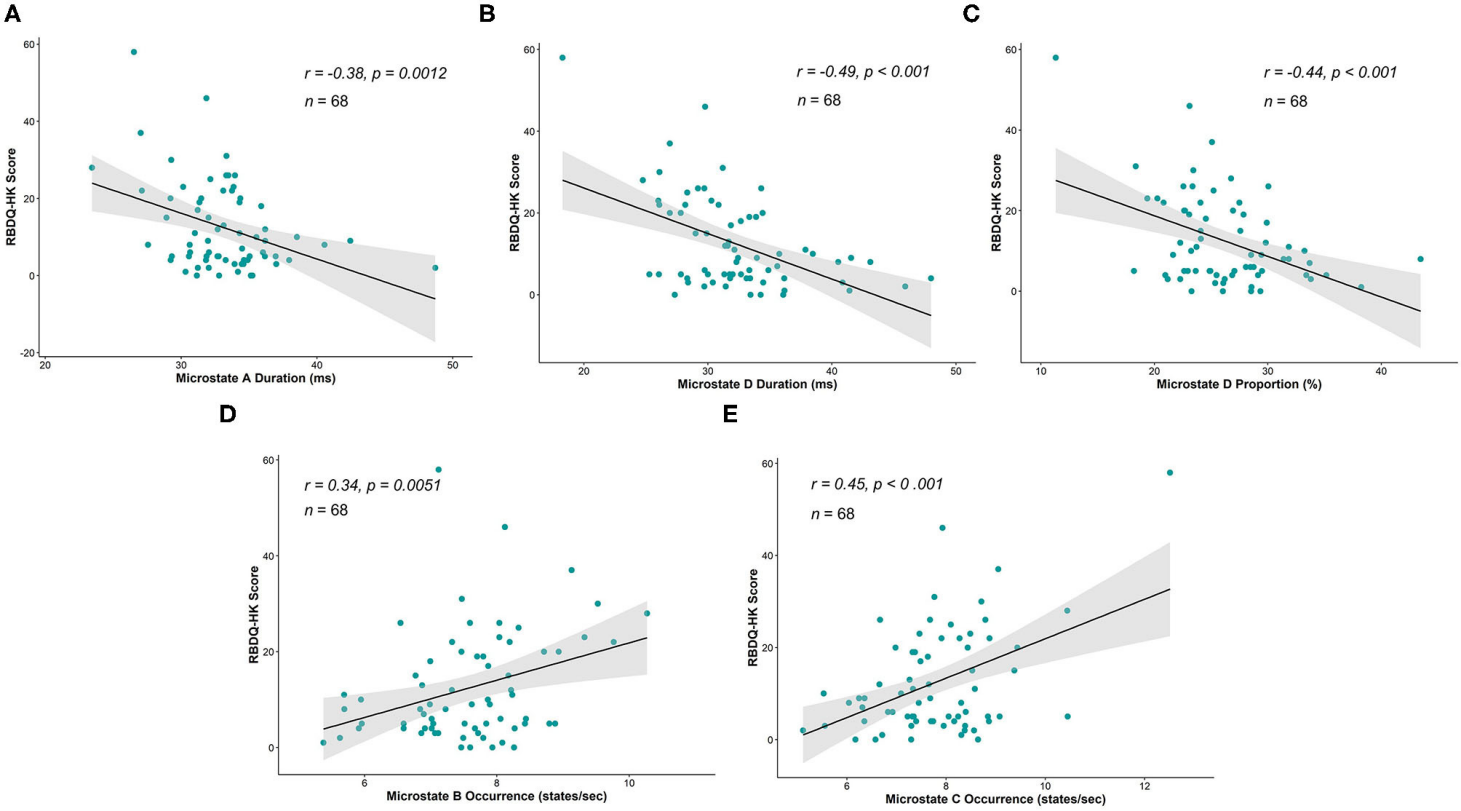
Correlations between microstate parameters and RBDQ-HK score. The average duration of microstate A (*r* = −0.38, *p* = 0.0012), average duration of microstate D (*r* = −0.49, *p* < 0.001), and proportion of microstate D (*r* = −0.44, *p* < 0.001) were significantly negatively correlated with the RBDQ-HK score **(A–C)**, while the occurrence of microstate B (*r* = 0.34, *p* = 0.0051) and occurrence of microstate C (*r* = 0.45, *p* < 0.001) were significantly positively related to the RBDQ-HK score **(D,E)**.

We further explored the relationship between the microstate parameters and the RBDQ-HK score. The correlational analysis that included all the individuals from both groups showed that the average duration, proportion of microstate D, and average duration of microstate A were negatively correlated with the RBDQ-HK score (*r* = −0.49, *p* < 0.001; *r* = −0.44, *p* < 0.001; and *r* = −0.38, *p* = 0.0012, respectively), while the occurrence of microstate B and microstate C was positively correlated with the RBDQ-HK score (*r* = 0.34, *p* = 0.0051 and *r* = 0.45, *p* < 0.001, respectively) (Figure 3).

**Figure 4:**
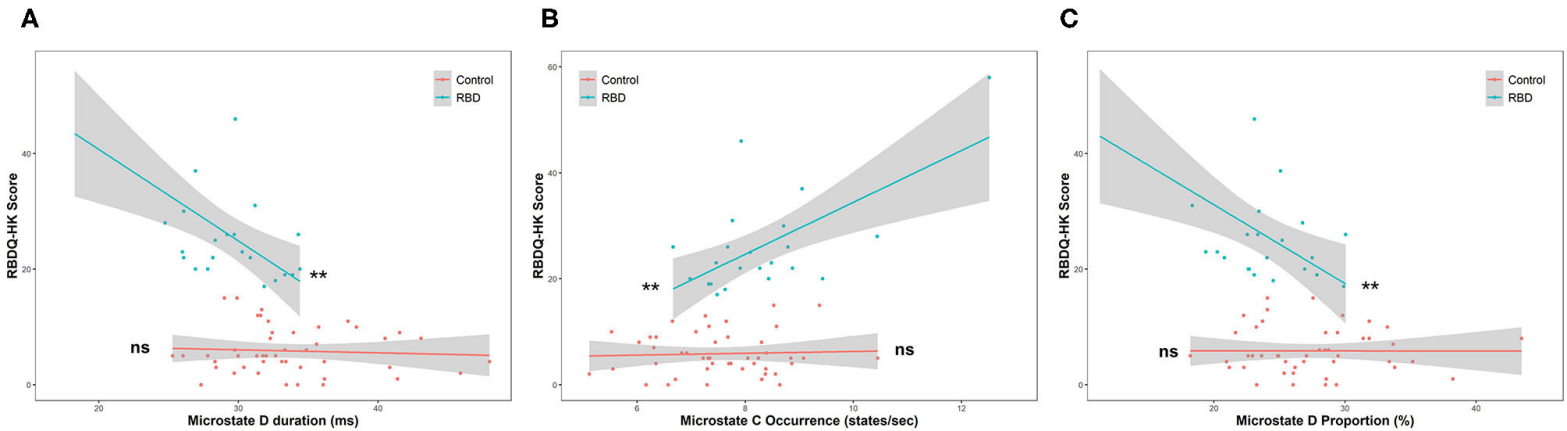
Linear regression between microstate parameters and RBDQ-HK score. The duration (β = −1.58, *p* = 0.0023) and proportion of microstate D (β = −1.36, *p* = 0.0048) were negatively associated with the RBDQ-HK score within the RBD group but not within the HC group (*p* = 0.67 and *p* = 0.99, respectively) **(A,B)**. The occurrence of microstate C (β = 4.90, *p* = 0.0014) was positively associated with the RBDQ-HK score within the RBD group but not within the HC group (*p* = 0.76) **(C)**. ns, not significant; **p < 0.01.

Moreover, linear regression was implemented within the two groups to further test the association between the parameters and RBD severity. After testing the statistical assumptions of linear regression, the duration and proportion of microstate D and the occurrence of microstates B and C were valid, and the RBDQ-HK score was regressed on them. The results showed that the duration (β = −1.58, *p* = 0.0023) and proportion (β = −1.36, *p* = 0.0048) of microstate D were negatively associated with the RBDQ-HK score within the RBD group but not within the HC group (*p* = 0.67 and *p* = 0.99). The significant ANCOVA test (both *p* < 0.001) further revealed the effect of grouping on the relationship between these microstate parameters and RBDQ-HK score. The occurrence of microstate C (β = 4.90, *p* = 0.0014) was positively associated with the RBDQ-HK score within the RBD group but not within the HC group (*p* = 0.76). The ANCOVA test also revealed a positive result (*p* < 0.001). The occurrence of microstate B was not associated with the RBDQ-HK score neither in the RBD group (*p* = 0.83) nor in the HC group (*p* = 0.84) (Figure 4).

### Microstate Transitions Were Altered in the RBD Group

**Figure 5:**
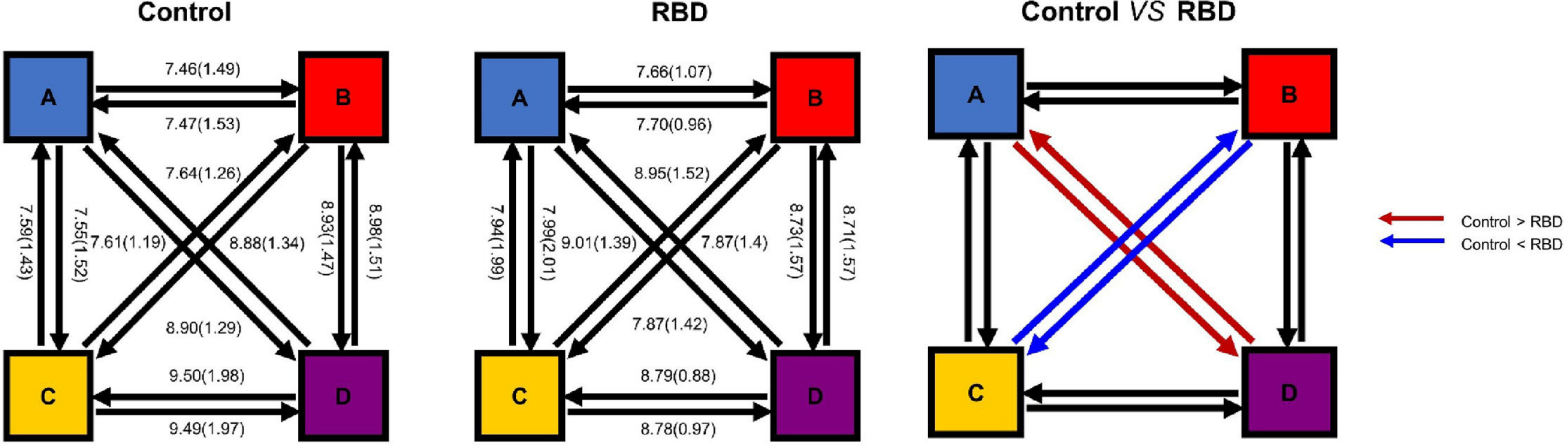
Microstate transitions were altered in the RBD group. The mean observed percentage of transitions of HCs **(Left)** and participants with RBD **(Middle)**. The frequencies of transitions from microstate A to D and D to A were significantly decreased in the RBD group, while the RBD group produced increased frequencies of transitions between B and C **(Right)**.

Finally, the percentages of transitions were compared with the expected value by using unpaired *t*-test. The result showed that the observed transition was similar to the expected value in both groups. The plot of transition with mean and SE was shown in Figure 5. Furthermore, we compared the observed transitions between two groups, and the result showed that the frequencies of transitions from microstates A to D and D to A were significantly decreased in the RBD group than the HCs (*t* = −2.87, *p*_adjusted_ = 0.0066, Cohen's *d* = −0.77 and *t* = −2.81, *p*_adjusted_ = 0.0074, Cohen's *d* = −0.74, respectively), while the RBD group produced increased frequencies of transitions from microstate B to C and C to B (*t* = 3.52, *p*_adjusted_ = 0.0012, Cohen's *d* = 0.97 and *t* = 4.05, *p*_adjusted_ < 0.001, Cohen's *d* = 1.11, respectively) (Figure 5).

## Discussion

In this study, we found that the topological structure of the brain and the temporal characteristics of the sub-second brain activity were significantly altered in RBD, which cast a new light on the abnormality of the electrophysiological features of RBD. Furthermore, we found that several parameters of EEG microstates were closely related to the RBDQ-HK score. These results indicated that abnormalities of resting-state EEG microstates may be endophenotypes of RBD with a great potential clinical value.

The results showed a significant alteration of the topological structure in RBD, which was characterized by the more lateralization and less contribution of microstate D. The alteration of spatial topography of EEG microstate D shown in RBD was also found in PD (Chu et al., 2020), which was believed to be due to the deficit of dopaminergic neurons (Van den Brink et al., 2018). The previous study also found that the frequency and duration of microstate D were significantly increased after taking levodopa, suggesting that the decreased duration of microstate D reflects abnormal dopaminergic activity (Serrano et al., 2018). Interestingly, we found that the average duration and proportion of microstate D were negatively correlated with the RBDQ-HK score, which represents the clinical RBD severity (Li et al., 2010). The results of this study, together with previous findings, demonstrated that the alteration of microstate D parameters could represent the degree of dopaminergic deficit.

Considering topography, microstate D was believed to be due to the activation of right inferior parietal, right middle and superior frontal gyri, and right insula (Custo et al., 2017). These areas were found to be vital for motor planning, executive control, and vision-guided movements (Kann et al., 2020). The alteration of the topological structure in microstate D implies the dysfunction of these areas in individuals with RBD. A similar finding was also found in patients with PD (Chen et al., 2021). What is more, the dysfunction of the functional connectivity of frontal-parietal- temporal network in individuals with RBD was also confirmed by the unusual transitions between microstates A and D. The deviated pattern may further indicate an imbalance between the resting state network and a deficit of several cognition processes. Taken together, these studies suggest that the resting-state EEG microstate D could reflect early abnormality of the frontal-parietal network, which might be critical for the pathological mechanism of neurodegenerative diseases and could be detected long before the occurrence of movement symptoms. Specifically, the decreased duration of microstate D has been reported in affective (Murphy et al., 2020) and psychotic disorders (Rieger et al., 2016). These findings may suggest that microstate D may be involved in the common pathophysiological process of many neuropsychiatric diseases.

Other disruption patterns of microstate dynamics including the increased frequency of microstate B and increased transitions between microstates B and C were also found in RBD, which could indicate the aberrant functional connectivity between the salience network and the visual cortex. Since the anterior insular receives the convergent multisensory inputs and regulates cognition (Menon and Uddin, 2010), the disturbance of microstate dynamics indicates a disturbance of these areas.

There are some limitations in this study. First, all patients with RBD were diagnosed by questionnaires and clinical evaluation. Therefore, the confirmation of these results in a group of patients with RBD diagnosed with the “gold standard”- polysomnography is warranted. Second, this study is unable to localize the corresponding neural correlate of each microstate status and the potential structural or functional connectivity abnormalities. Third, most participants belong to the aging group, which is unable to provide an indicator that tracks the development of RBD, and future longitudinal studies should be conducted to track the high-risk population and to reveal the underlying neural mechanism of the developmental trajectory of RBD.

## Conclusion

The results of this study deepened our understanding of brain dysfunction in the early stage of alpha-synucleinopathy diseases and suggested that microstates may be potential biomarkers of RBD. These results highlighted the clinical value of microstate analysis, which could enable early detection and diagnosis of RBD and early intervention accordingly.

## Data Availability Statement

The raw data supporting the conclusions of this article will be made available by the authors, without undue reservation.

## Ethics Statement

The studies involving human participants were reviewed and approved by the Ethics Committee of West China Hospital, Sichuan University. The participants provided their written informed consent to participate in this study.

## Author Contributions

AP: conceptualization, data curation, methodology, investigation, and writing—original draft. RW: formal analysis, investigation, methodology, and writing—original draft. JH: data curation, methodology, and writing—original draft. HW and LC: funding acquisition, resources, supervision, conceptualization, writing—review, and editing. All authors contributed to the article and approved the submitted version.

## Funding

This study was supported by 1·3·5 project for disciplines of excellence–Clinical Research Incubation Project, West China Hospital, Sichuan University (No. 2021HXFH012), the National Clinical Research Center for Geriatrics, West China Hospital, Sichuan University West China Hospital, Sichuan University (No. Z2018B24), and Shenzhen-Hong Kong-Macau Technology Research Programme (202011033000116).

## Conflict of Interest

The authors declare that the research was conducted in the absence of any commercial or financial relationships that could be construed as a potential conflict of interest.

## Publisher's Note

All claims expressed in this article are solely those of the authors and do not necessarily represent those of their affiliated organizations, or those of the publisher, the editors and the reviewers. Any product that may be evaluated in this article, or claim that may be made by its manufacturer, is not guaranteed or endorsed by the publisher.
